# Pelvic tilt after Bernese periacetabular osteotomy—a long-term follow-up study

**DOI:** 10.1093/jhps/hnad030

**Published:** 2023-09-09

**Authors:** Alexander F Heimann, Iris F Brouze, Guoyan Zheng, Angela M Moosmann, Joseph M Schwab, Moritz Tannast, Corinne A Zurmühle

**Affiliations:** Department of Orthopaedic Surgery and Traumatology, HFR—Cantonal Hospital, Chemin des Pensionnats 2-6, Fribourg 1700, Switzerland; Department of Medicine, University of Fribourg, Chemin du Musée, Fribourg 1700, Switzerland; Department of Orthopaedic Surgery, Valais Hospital, Avenue Grand-Champsec 80, Sitten 1951, Switzerland; Institute of Medical Robotics, School of Biomedical Engineering, Shanghai Jiao Tong University, Minhang District, 东川路 邮政编码, Shanghai 200240, China; Department of Orthopaedic Surgery and Traumatology, HFR—Cantonal Hospital, Chemin des Pensionnats 2-6, Fribourg 1700, Switzerland; Department of Medicine, University of Fribourg, Chemin du Musée, Fribourg 1700, Switzerland; Department of Orthopaedic Surgery and Traumatology, HFR—Cantonal Hospital, Chemin des Pensionnats 2-6, Fribourg 1700, Switzerland; Department of Orthopaedic Surgery and Traumatology, HFR—Cantonal Hospital, Chemin des Pensionnats 2-6, Fribourg 1700, Switzerland; Department of Medicine, University of Fribourg, Chemin du Musée, Fribourg 1700, Switzerland; Department of Orthopaedic Surgery and Traumatology, HFR—Cantonal Hospital, Chemin des Pensionnats 2-6, Fribourg 1700, Switzerland; Department of Medicine, University of Fribourg, Chemin du Musée, Fribourg 1700, Switzerland

## Abstract

Patients with developmental dysplasia of the hip (DDH) are believed to present with increased anterior pelvic tilt to compensate for reduced anterior femoral head coverage. If true, pelvic tilt in dysplastic patients should be high preoperatively and decrease after correction with periacetabular osteotomy (PAO). To date, the evolution of pelvic tilt in long-term follow-up after PAO has not been reported. We therefore asked the following questions: (i) is there a difference in pelvic tilt between patients with DDH and an asymptomatic control group? (ii) How does pelvic tilt evolve during long-term follow-up after Bernese PAO compared with before surgery? This study is a therapeutic study with the level of evidence III. We retrospectively compared preoperative pelvic tilt in 64 dysplastic patients (71 hips) with an asymptomatic control group of 20 patients (20 hips). In addition, immediate postoperative and long-term follow-up (at 18 ± 8 [range 7–34 years) pelvic tilt was assessed and compared. Dysplastic patients had a significantly higher mean preoperative pelvic tilt than controls [2.3 ± 5.3° (−11.2° to 16.4°) versus 1.1 ± 3.0° (−4.9 to 5.9), *P* = 0.006]. Mean pelvic tilt postoperatively was 1.5 ± 5.3° (−11.2 to 17.0º, *P* = 0.221) and at long-term follow-up was 0.4 ± 5.7° (range −9.9° to 20.9°, *P* = 0.002). Dysplastic hips undergoing PAO show a statistically significant decrease in pelvic tilt during long-term follow-up. However, given the large interindividual variability in pelvic tilt, the observed differences may not achieve clinical significance.

## INTRODUCTION

### Background

In developmental dysplasia of the hip (DDH), the location of femoral head coverage deficiency demonstrates spatial variability [1]. However, the most common location for coverage deficiency is the anterolateral femoral head [2]. In combination with an undersized lunate surface in dysplastic hips [3], patients experience inadequate load transfer from the acetabulum to the femur with joint instability. If left untreated, this leads to hip pain, cartilage degeneration and premature development of osteoarthritis [4–7].

To compensate for reduced femoral head coverage and to improve load transfer in the hip joint, some authors have hypothesized that patients with DDH reactively tilt their pelvis more anteriorly [8, 9]. If true, pelvic tilt should be high in dysplastic patients and subsequently decrease after surgical correction of femoral head coverage via periacetabular osteotomy (PAO) [10].

### Rationale

To date, there is limited evidence on both pelvic tilt in dysplastic hips preoperatively, as well as its evolution following PAO with no available data on the long-term evolution. In addition, studies mainly use 2D measurement methods on anteroposterior (AP) pelvis radiographs [10–12]. To the best of our knowledge, this is the first study to analyze the long-term evolution of pelvic tilt following PAO using a novel, previously validated 2D–3D deformation–reconstruction software for improved analysis of the 3D pelvic orientation [13].

We therefore asked the following questions: (1) is there a difference in pelvic tilt between patients with DDH and an asymptomatic control group? (2) How does pelvic tilt evolve in the long-term course after Bernese PAO compared with before surgery?

## MATERIALS AND METHODS

### Study design and settings

This was an institutional review board–approved, single-center, retrospective, longitudinal controlled study conducted at a tertiary center for joint preservation surgery. We analyzed preoperative, postoperative and long-term follow-up AP pelvis radiographs using the previously validated software program HipRecon [13]. The analysis included a comparison of preoperative pelvic tilt with an asymptomatic control group as well as measurement of pelvic tilt immediately following PAO and during long-term follow-up.

### Participants

**Fig. 1.**
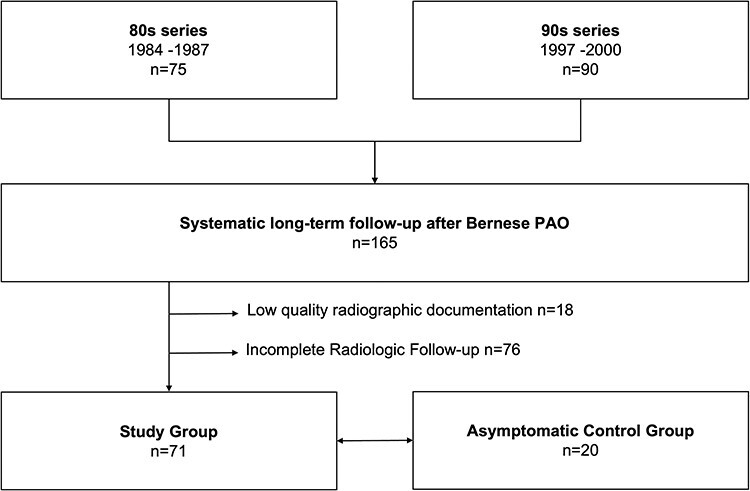
Study flowchart.

We performed a retrospective review of two previously published series of patients who underwent PAO with the technique described by Ganz *et al.* [14] at the inventor’s institution in the 1980s [15] and 1990s [16]. Of a total of 147 patients (165 hips), we subsequently excluded 18 patients (18 hips) for having low-quality radiographic documentation and 65 patients (76 hips) for an incomplete radiologic follow-up, resulting in a study group of 64 patients (71 hips). Of those, 45 were women (70%) and 32 were right hips (50%). The mean age at surgery was 29 ± 9 years. The study group was then compared with a control group of 20 asymptomatic patients (20 hips; Fig. 1).

### Experimental setup

At our Department of Orthopaedic Surgery and Traumatology at HFR - Cantonal Hospital , AP pelvis radiographs are obtained according to a previously described, standardized acquisition technique [17]. Briefly, patients are in the supine position with legs 15° internally rotated to compensate for femoral antetorsion. The film-focus distance is 1.2 m with the central beam directed to the midpoint between a line connecting both anterior superior iliac spines (ASIS) and the pubic symphysis.

**Fig. 2.**
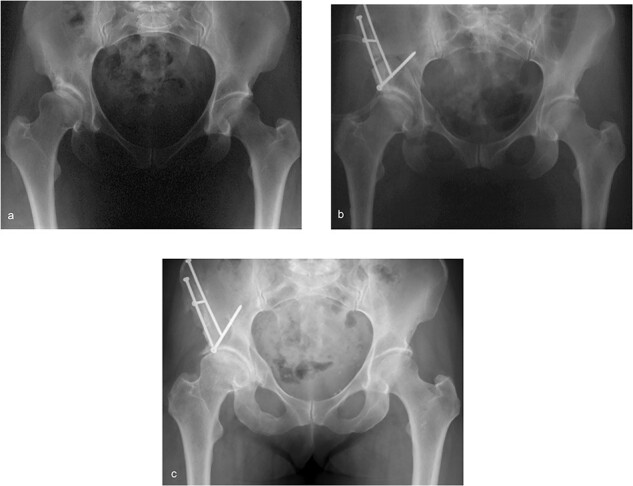
(a) Preoperative, (b) postoperative and (c) 10-year follow-up AP pelvis radiographs of a female patient with DDH who underwent periacetabular reorientation osteotomy.

Preoperative, postoperative and follow-up AP pelvis radiographs were available in the institution’s picture archiving and communication system for all included patients. If multiple follow-up images were available, we always chose the AP pelvis radiograph with the greatest time interval since surgery. In that way, we selected a total of 213 AP pelvis radiographs for further evaluation (Fig. 2). Mean radiographic follow-up was 18 ± 8 years (range 7–34) postoperatively.

### HipRecon

**Fig. 3.**
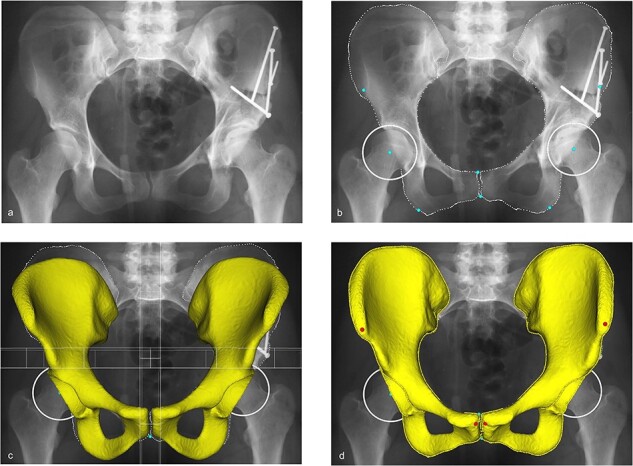
HipRecon workflow. (a) A standardized AP pelvis radiograph is uploaded. (b) Manual segmentation of the outer osseous border of the pelvis is performed, and (c) a standard 3D pelvis model is overlaid. The software’s 2D–3D deformation–reconstruction algorithm then transforms this model into a (d) patient-specific 3D pelvis model allowing for calculation of pelvic tilt with respect to the APP.

HipRecon is a software that has been previously validated [13] and uses a statistical shaped model based on a 2D to 3D deformation–reconstruction method to create a patient-specific 3D model based solely on an AP pelvis radiograph (Fig. 3). This virtual 3D model allows for accurate, precise and reliable computation of pelvic tilt with respect to the anterior pelvic plane (APP). The APP is defined as the plane between a line connecting the ASIS and the pubic tubercles. According to the definition of the Hip-Spine workgroup, anterior (positive) pelvic tilt is defined as an anterior rotation of the ASIS with respect to the pubic tubercles, while posterior (negative) pelvic tilt is defined as a posterior rotation of the ASIS with respect to the pubic tubercles [18].

### Pelvic tilt in dysplastic patients compared with an asymptomatic control group

All 71 preoperative AP pelvis radiographs of the dysplastic hips were analyzed using HipRecon. The mean preoperative pelvic tilt in these dysplastic hips was then compared with the mean pelvic tilt in a control group of 20 asymptomatic patients (20 hips) who had undergone pelvic computed tomography scans for non-orthopedic reasons. Directly reconstructed radiographs were created from the scans and subsequently analyzed using HipRecon.

### Evolution of pelvic tilt after PAO

To assess the evolution of pelvic tilt after PAO during long-term follow-up, the immediate postoperative as well as long-term follow-up AP pelvis radiographs of the patients who underwent Bernese PAO were analyzed using HipRecon. The determination of pelvic tilt values at the three different time points (preoperative, postoperative and at long-term follow-up) permitted the analysis of any significant changes in pelvic tilt over time following PAO.

### Statistical analysis

We performed statistical analysis using a commercially available add-in for Microsoft Excel (Winstat©, R. Fitch Software, Germany) and MedCalc® Statistical Software version 20.106 (MedCalc Software Ltd, Ostend, Belgium). After distribution testing with the Kolmogorov–Smirnov test, normally distributed data were analyzed using a repeated-measures analysis of variance, and non-normally distributed data were analyzed using the Friedman test. The significance level was set at α = 0.05, and thus, a statistical test was considered significant if *P* < 0.05.

## RESULTS

### Difference in pelvic tilt between patients with dysplastic hips and an asymptomatic control group

**Fig. 4.**
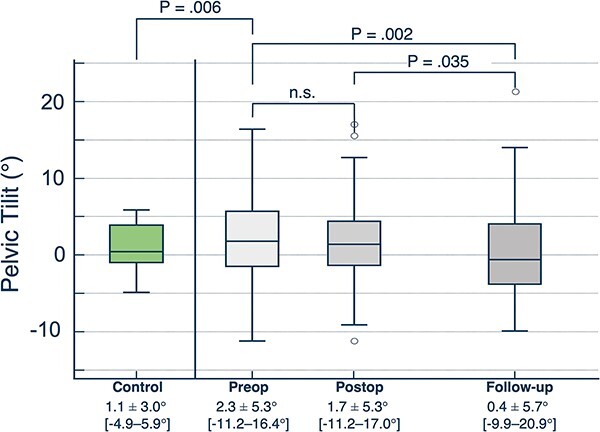
Pelvic tilt (in °) of the asymptomatic control group and dysplastic patients preoperatively (Preop), as well as after Bernese PAO (Postop) and at long-term follow-up (Follow-up). Boxplots are depicted as the boxes being the first quartile, median and third quartile, and the whiskers representing the minimum and maximum value observed. n.s. = not significant.

Patients with dysplastic hips had a significantly higher pelvic tilt preoperatively compared with the asymptomatic control group [2.3 ± 5.3° (−11.2 to 16.4) versus 1.1 ± 3.0° (−4.9 to 5.9), *P* = 0.006; Fig. 4]. The difference in means of 1.2°, while statistically significant, was smaller than the observed standard deviations (5.3º for the preoperative group and 3.0º for the control group).

### Long-term evolution of pelvic tilt following Bernese PAO

Postoperatively, the mean pelvic tilt was 1.5 ± 5.3° (−11.2 to 17.0; *P* = 0.221), and at long-term follow-up, the mean pelvic tilt was 0.4 ± 5.7° (−9.9 to 20.9). In comparison to both the preoperative (*P* = 0.002) and immediate postoperative (*P* = 0.035) pelvic tilt, the observed change was statistically significant (Fig. 4), but we again observed that the difference in means of 1.9º (preoperative to long term) and 1.1º (postoperative to long term) was less than the standard deviations for each group (5.3º for the preoperative group, 5.3º for the postoperative group and 5.7º for the long-term follow-up group).

## DISCUSSION

The specific role of pelvic tilt in the pathophysiology of DDH has not yet been completely elucidated [12] and is the subject of ongoing clinical research. To the best of our knowledge, no long-term results have been published on the evolution of pelvic tilt after PAO. With available access to a large cohort of patients with long-term follow-up after PAO at the inventor’s institution, we therefore asked the following questions: (1) is there a difference in pelvic tilt between patients with DDH and an asymptomatic control group? (2) How does pelvic tilt evolve in the long-term course after Bernese PAO compared with before surgery?

At a mean follow-up of 18 ± 8 years (range 7–34 years), there was a significant (*P* = 0.002) decrease in pelvic tilt from 2.3 ± 5.3° (range −11.2 to 16.4) preoperatively to 0.4 ± 5.7° (range −9.0 to 20.9) at long-term follow-up.

It has been hypothesized that patients with DDH increase their lumbar lordosis, and thus pelvic tilt, to compensate for the lack of femoral head coverage in the weight-bearing zone [8, 9, 19]. If this assumption is correct, it would be logical to observe a decrease in pelvic tilt after surgical improvement of superolateral femoral head coverage through PAO. The investigation of this hypothesis is the subject of current clinical research (Table I). When comparing the results of this study with other reported findings, several methodological differences should be considered. Only Tani *et al.* [20] used the APP as a reference for measuring pelvic tilt, which is the same measurement methodology as HipRecon. All other authors who investigated the change in pelvic tilt after acetabular reorientation osteotomy used either the sacro-femoral-pubic angle [21] or the pubic symphysis to sacroiliac index [10]. These two measurement methods showed a lower correlation with actual pelvic tilt compared with HipRecon [13].

**Table I.** Summary of reported findings on the effect of acetabular reorientation osteotomy on pelvic tilt in patients with DDH

*Author*	*Year*	*No. of hips (patients*)	*Surgical intervention*	*Mean follow-up (years*)	*Tilt measurement via*	*Significant change in PT*
Daley *et al*. [10]	2019	40 (40)	Staged bilateral PAO	1.3 ± 0.5	PS-SI	Yes
Roussot *et al*. [12]	2020	64 (48)	Uni- and bilateral PAO	2.6 ± 1	SFP and PS-SI	No
Grammatopoulos *et al*. [11]	2020	48 (42)	Anteverting PAO	2.5 ± 2	SFP	No
Tani *et al.* [20]	2020	25 (25)	RAO or CPO	2	PSI	Yes

PT = pelvic tilt; RAO = rotational acetabular osteotomy; CPO = curved acetabular osteotomy; SFP = sacro-femoral-pubic; PS-SI = pubic symphysis to sacroiliac index; PSI = pelvic sagittal inclination.

The total number of patients included in the comparable studies was slightly smaller than that in our study. In addition, they partially considered different surgical techniques [20] or even different surgical indications [11] compared with the present study. Another relevant difference and unique feature of our study is the significantly longer follow-up period of 18 ± 8 years on average. Despite these relevant methodological differences, the main findings of these studies are comparable to ours. A possible explanation for the fact that some authors did not find a significant decrease could be related to the precision of measurement methods used or to the heterogeneity of their study cohorts.

In light of our findings and in agreement with other authors [11, 12, 20, 22], while we observe a statistically significant decrease in pelvic tilt after PAO over the long term, we also observe a large interindividual difference in pelvic tilt, both before and after PAO. Given this variability, it is reasonable to question whether the absolute mean change of two to three degrees reported in the literature, and confirmed in our study, is clinically relevant.

One approach to the issue of questionable clinical relevance is the use of a ‘minimal clinically important difference’ (MCID). Different methods have been described to define the MCID [23], one of which is the distribution-based approach. The idea behind this approach is that a clinically relevant difference should be at least as large as 1 SD of the measured values. Given the high interindividual variability of pelvic tilt, distribution-based MCID may be a reasonable approach to evaluate the clinical relevance of any changes in pelvic tilt. Using this method raises further concern that the observed statistically significant changes in pelvic tilt following PAO do not reach the level of clinical significance.

This study had several limitations. First, we only performed a static analysis of pelvic tilt, and no dynamic assessment was performed. In addition, we did not take sacral morphometric parameters into account. However, the reference values in current use for the assessment of pelvis and acetabular morphology are based on supine pelvis radiographs. Furthermore, the standardized acquisition technique of these radiographs enhances the comparability of our findings. Second, the time span of follow-up was quite wide, with an absolute difference of 27 years between the earliest and latest follow-ups. Nevertheless, the minimum follow-up was 8 years postoperatively, which we feel represents a reasonable long-term follow-up. Third, the natural history of lumbar kyphosis and its possible influence on pelvic tilt were not considered. It has been reported that patients exhibit a decrease in lumbar lordosis over time, which could partially explain the long-term decrease in pelvic tilt we observed. However, no patients complained of lower back pain or had undergone spinal surgery by the time of long-term follow-up. Fourth, the retrospective study design demonstrates inherent shortcomings such as the inability to recognize confounding variables. Despite this potential shortcoming, we feel the data and analysis presented remain valuable in an area that has remained largely under-explored.

Patients with dysplastic hips undergoing PAO have significant variability in their preoperative pelvic tilt but see a statistically significant decrease in pelvic tilt during long-term follow-up after PAO. This observed decrease may support the hypothesis that dysplastic hips undergoing PAO can ‘normalize’ their pelvic tilt once coverage is improved. On the other hand, however, given the large interindividual variability in pelvic tilt, the observed differences in pelvic tilt may not reach the threshold for clinical importance.

## Data Availability

The data underlying this article will be shared on reasonable request to the corresponding author.
